# Sildenafil treatment attenuates ventricular remodeling in an experimental model of aortic regurgitation

**DOI:** 10.1186/s40064-015-1317-8

**Published:** 2015-10-13

**Authors:** Kristian Eskesen, Niels Thue Olsen, Veronica L. Dimaano, Thomas Fritz-Hansen, Peter Sogaard, Khalid Chakir, Charles Steenbergen, David Kass, Theodore P. Abraham

**Affiliations:** Johns Hopkins Medical Institutions, Division of Cardiology, Baltimore, MD USA; Department of Cardiology, Gentofte Hospital, University of Copenhagen, Niels Andersensvej 65, 2900 Hellerup, Denmark; Department of Cardiology, Aalborg Hospital, University of Aalborg, Aalborg, Denmark

**Keywords:** Aortic regurgitation, Echocardiography, Animal models, Phosphodiesterase

## Abstract

**Background:**

Currently there is no reliable medical treatment for aortic regurgitation (AR).

**Methods:**

Thirty-nine Sprague–Dawley rats underwent creation of AR or sham operation. Treated rats were assigned to early or late institution of sildenafil therapy (100 mg/kg/day) for a total of 10 weeks. Treatment–effects were measured by serial echocardiography, invasive hemodynamic measurements, and tissue analysis.

**Results:**

Rats assigned to early treatment developed less remodeling than untreated rats. Thus, left ventricular (LV) dilation was blunted by sildenafil with end–systolic diameter being significantly smaller (6.6 ± 0.4 vs. 7.7 ± 0.4 mm, respectively, p < 0.05). Also, LV wall thickness was significantly decreased in treated rats compared to controls (2.23 ± 0.08 vs. 2.16 ± 0.05 mm, p < 0.01). Fractional shortening was improved by treatment (p < 0.05). Myocardial fibrosis was borderline decreased by treatment (p = 0.09). Akt was increased in treated compared to controls (p < 0.05).

**Conclusion:**

Sildenafil slightly inhibits LV remodeling and improves fractional shortening in rats with AR when treatment is initiated early.

## Background

Aortic regurgitation (AR) is a common valvular disease that affects men more than women, and whose incidence increases with age (Maurer 2006). Severe AR is associated with higher morbidity and mortality compared to the general population (Dujardin et al. 1999). Chronic AR results in a mixed hypertrophy phenotype (eccentric and concentric) resulting from predominantly volume but also elements of pressure overload (Opie et al. 2006). Although well tolerated for years, persistent overload is associated with activation of humoral systems such as the renin-angiotensin and sympathetic nervous system leading to myocyte hypertrophy resulting in a dilated left ventricle (LV) and systolic dysfunction. Once symptoms or systolic dysfunction in asymptomatic patients occur, outcomes become worse. However, if intervention is performed early, LV damage appears to be reversible (Bonow et al. 1984, 1985; Dujardin et al. 1999). Aortic valve replacement (AVR) is the most definitive treatment and LV dimensions act to be good indicators for timing of surgery and predictors of outcomes post surgery. Over decades, several attempts have been made to determine the effect of different pharmacologic therapies (Lin and Stewart 2011). However, there are no large randomized clinical trials, and there is substantial inconsistency in the results of published studies (Søndergaard et al. 2000; Evangelista et al. 2005). As a result the American Heart Association/American College of Cardiology and European Society of Cardiology offer only class IIb (level of evidence B) recommendations for vasodilator therapy in AR (Vahanian et al. 2012; Nishimura et al. 2014). Phosphodiesterase 5A (PDE5A) inhibition has demonstrated positive cardiac effects in various disease models, including mitral valve regurgitation (Kim et al. 2012). Recently, PDE5A-inhibition has shown to abrogate cardiac remodeling and deterioration of LV function in pressure overload by inhibiting several pro–hypertrophic signaling pathways (Takimoto et al. 2005). Yet, the effects of PDE5A-inhibition in AR are unknown. We examined the effects of PDE5A-inhibition in a rodent model of chronic AR with respect to LV remodeling and LV function, particularly clarifying the more clinical applicable question of timing of therapy vis-à-vis disease duration. Additionally, we investigated if the effects on the heart were mediated by molecular mechanisms known to be operative in pressure overload hypertrophy.

## Results and discussion

Three rats died during the study–period; one from the AR (early) group, one from the AR + SIL (late) group and one from the AR (late) group. Only data from surviving rats were included in the final analysis.

### Morphology and function

**Fig. 1:**
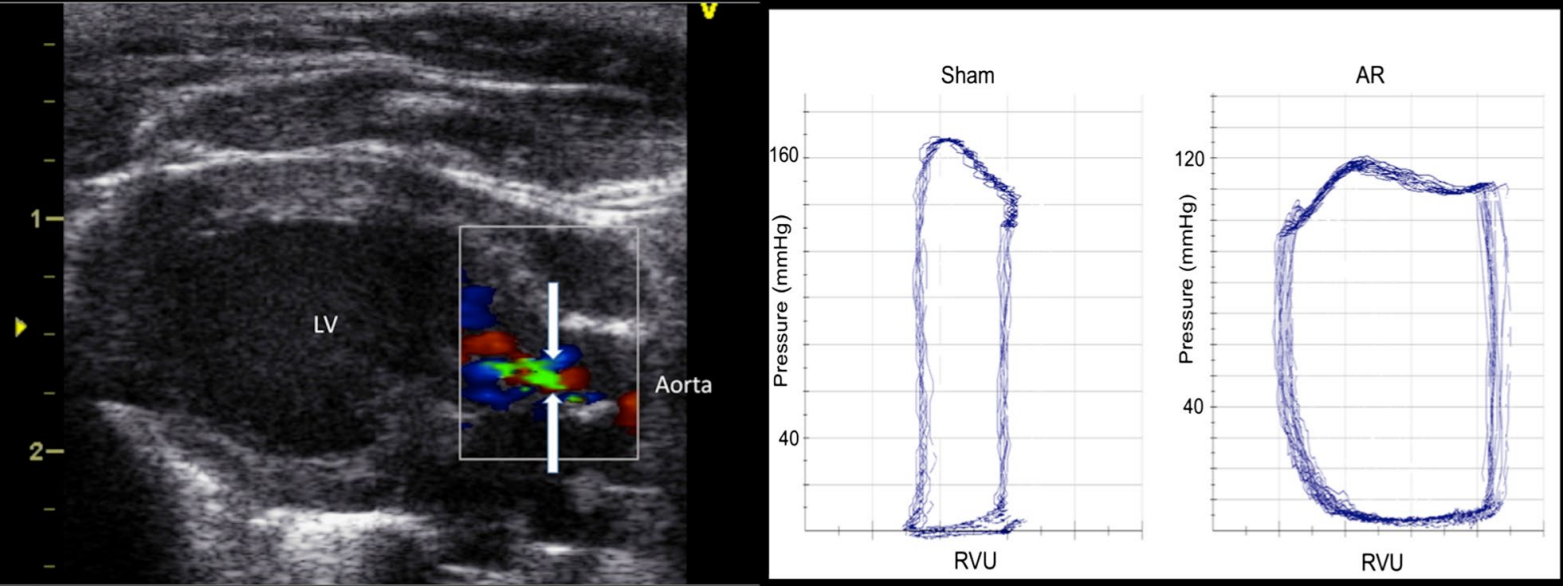
Aortic regurgitation. *Left panel* shows left ventricle (LV) from a rat with aortic regurgitation with regurgitation indicated by *arrows*. *Right panel* shows pressure volume loops from a sham-operated (*left*) and untreated (AR) rat (*right*). The *horizontal axis* indicate relative volume unit (RVU) and the *vertical axis* indicate intraventricular pressure

AR resulted in classic eccentric hypertrophy and led to rightward shift of PV–loops indicating LV remodeling, Fig. 1. Table 1 shows echocardiographic characteristics at baseline and 2 weeks after AR–induction. Table 2 shows morphometric measures at sacrifice.

**Table 1:** Echocardiographic characteristics

	Baseline		Two weeks	
	Sham (n = 8) Mean	AR (n = 8) Mean	Sham (n = 8) Mean	AR (n = 8) Mean
IVSd	1.54 ± 0.06	1.55 ± 0.06	1.68 ± 0.03	1.85 ± 0.06*
PWd	1.99 ± 0.05	2.04 ± 0.05	1.94 ± 0.04	2.13 ± 0.06*
LVEDD	8.66 ± 0.08	9.08 ± 0.09	8.90 ± 0.16	10.36 ± 0.23*
LVESD	5.16 ± 0.10	5.21 ± 0.18	5.09 ± 0.21	6.05 ± 0.30*
Strain	−23.11 ± 0.78	−22.66 ± 1.09	−23.26 ± 1.02	−22.52 ± 1.03
SR (systolic)	−5.43 ± 0.27	−5.31 ± 0.30	−5.33 ± 0.31	−5.12 ± 0.31
SR (diastolic)	6.41 ± 0.58	6.15 ± 0.38	4.41 ± 1.45	5.25 ± 0.32
FS	0.40 ± 0.01	0.43 ± 0.02	0.43 ± 0.02	0.42 ± 0.02

Intraventricular septum thickness in diastole (IVSd), posterior wall thickness in diastole (PWd), left ventricular end–diastolic diameter (LVEDD), left ventricular end–systolic diameter (LVESD), strain rate (SR), fractional shortening (FS). Measurements are given as mean (SEM)

* p < 0.05 (sham vs. AR)

**Table 2:** Morphometric measure at sacrifice

	Early					Late		
	Sham (n = 8)	AR + SIL (n = 8)	AR (n = 7)	p value (disease)	p value (treatment)	AR + SIL (n = 6)	AR (n = 5)	p value (treatment)
Body weight, g	670 ± 18	627 ± 13	711 ± 28	0.23	0.01*	657 ± 28	731 ± 53	0.23
Heart weight, g	1.66 ± 0.07	2.07 ± 0.13	2.45 ± 0.16	<0.05*	0.08	2.00 ± 0.11	2.27 ± 0.25	0.30
Heart weight ratio*, mg	2.49 ± 0.12	3.30 ± 0.17	3.47 ± 0.23	<0.01*	0.57	3.06 ± 0.21	3.08 ± 0.18	0.94
LV weight, g	1.24 ± 0.08	1.51 ± 0.09	1.76 ± 0.13	0.01*	0.13	1.49 ± 0.09	1.65 ± 0.17	0.40
RV weight, g	0.26 ± 0.01	0.30 ± 0.02	0.36 ± 0.03	<0.05*	0.14	0.31 ± 0.03	0.36 ± 0.04	0.34
Lung weight, g	1.59 ± 0.03	1.73 ± 0.04	2.10 ± 0.36	0.11	0.34	1.69 ± 0.07	1.72 ± 0.11	0.86

Comparison of morphometric measures at sacrifice between sham and AR (disease p value) and between AR and AR+SIL (treatment p value). Left ventricle (LV) and right ventricle (RV)

* p value < 0.05

### Early treatment

**Fig. 2:**
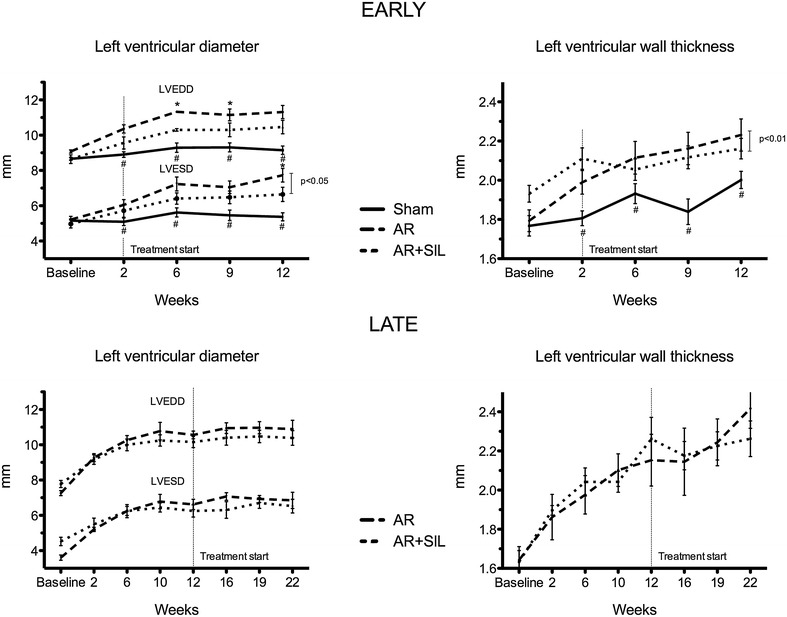
Left ventricle remodeling. LV diameters in end-diastole (LVEDD) and end–systole (LVESD) (*left panel*) and LV wall thickness (*right panel*) for early treated groups (*upper*) and late treated groups (*lower*). *Asterisk* indicate significant difference between AR vs. AR + SIL and *hash* indicate difference between sham vs. AR at individual time points (*t* test/ANOVA). *Vertical bar* and related p value is for overall difference (repeated measures)

Echocardiography showed AR + SIL (early) rats to develop less remodeling than AR (early). LV dimensions increased in all rats with AR, starting immediately after AR–induction. In AR (early) LV dilation continued throughout the study-period while treatment with sildenafil blunted this response, Fig. 2. LVEDD was significantly lower after six and 9 weeks but did not in overall analysis. LVESD was significantly lower in AR + SIL (early) compared to AR (early) after twelve weeks and in overall analysis (p = 0.03). Furthermore average LV wall thickness was significantly lower in AR + SIL (early) compared to AR (early) in overall analysis (p = 0.004) and consequently a lower LV mass was observed (p = 0.005, not shown).

**Fig. 3:**
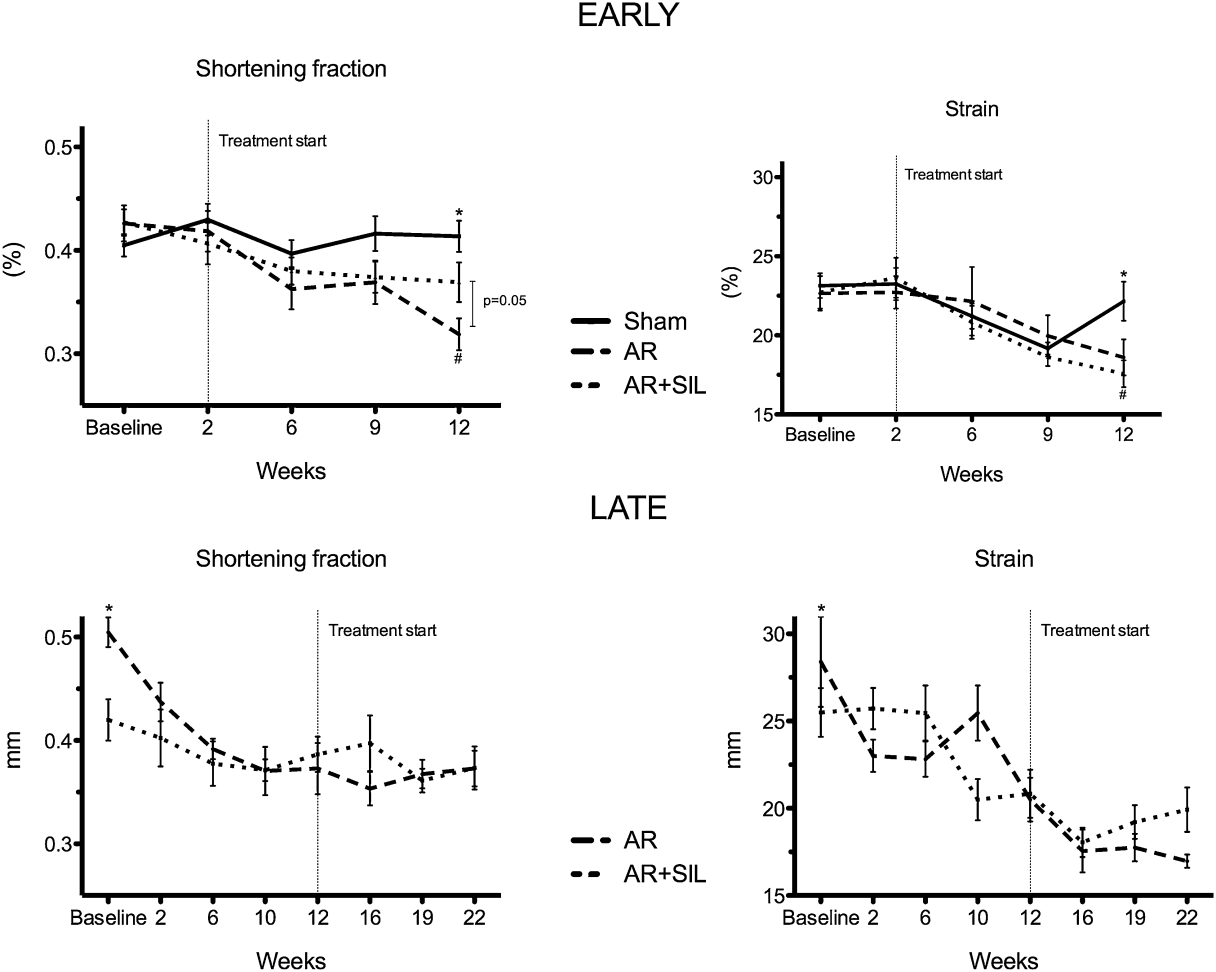
Left ventricle function. LV function in early (*upper panel*) and late (*lower panel*) treated groups measured by fractional shortening (FS) (*left*) and by speckle tracking (*right*). *Asterisk* indicate significant difference between AR vs. AR + SIL and *hash* indicate difference between sham vs. AR at individual time points (t test/ANOVA). *Vertical bar* and related p value is for overall difference (repeated measures)

As seen in Fig. 3 conventional echocardiographic measures of LV function showed less decrease in FS in AR + SIL (early) than in AR (early) after 12 weeks and in overall analysis (p = 0.01). We did not find any difference in LV performance measured by speckle tracking echocardiography.

In contrast, invasive hemodynamic measurements showed no differences in measurements of LV performance or filling between treated and untreated groups, Table 3. Pulse pressure (PP) was unaltered between AR (early) and AR + SIL (early).

**Table 3:** Invasive hemodynamic measurements

	Baseline			Sacrifice			
	Sham (n = 8)	AR (n = 8)	AR + SIL (n = 8)	Sham (n = 6)	AR (n = 4)	AR + SIL (n = 4)	p value
(A) Early treatment group							
HR	318 ± 14	316 ± 8	324 ± 18	355 ± 11	324 ± 8	333 ± 15	0.97
Systolic BP	133 ± 7	134 ± 6	134 ± 11	140 ± 4	140 ± 10	131 ± 8	1.00
Diastolic BP	90 ± 4	91 ± 4	98 ± 5	92 ± 2	78 ± 7	78 ± 2	0.57
PP	42 ± 10	46 ± 10	35 ± 11	47 ± 6	62 ± 7	53 ± 7	1.00
dp/dtmax	8243 ± 280	8841 ± 220	8645 ± 301	8150 ± 204	7440 ± 317	7864 ± 290	0.96
dp/dtmin (−)	6960 ± 318	7620 ± 240	7944 ± 421	7165 ± 461	5446 ± 432	5904 ± 617	1.00
LVESP	131 ± 10	137 ± 8	131 ± 11	129 ± 9	125 ± 12	126 ± 13	0.26
LVEDP	9.1 ± 1.3	8.1 ± 2.0	8.3 ± 1.2	8.1 ± 1.9	5.7 ± 2.4	9.8 ± 4.6	0.96
Tau	12.4 ± 0.7	12.4 ± 0.7	12.1 ± 0.6	13.8 ± 1.1	11.1 ± 2.1	13.3 ± 2.6	0.81

	Baseline			Sacrifice			
	AR (n = 5)	AR + SIL (n = 6)	AR (n = 5)	AR + SIL (n = 6)	p value		
(B) Late treatment group							
HR	296 ± 12	269 ± 12	235 ± 6	267 ± 10	0.02*		
Systolic BP	117 ± 3	115 ± 4	131 ± 6	128 ± 3	0.98		
Diastolic BP	85 ± 2	82 ± 3	65 ± 3	82 ± 2	0.03*		
PP	31 ± 2	33 ± 2	66 ± 7	46 ± 4	0.05*		
dp/dtmax	7600 ± 225	7810 ± 339	6473 ± 386	8211 ± 203	0.44		
dp/dtmin (−)	6836 ± 552	6893 ± 499	7120 ± 1474	6443 ± 556	0.25		
LVESP	104 ± 10	110 ± 7	148 ± 4	125 ± 9	0.29		
LVEDP	4.9 ± 0.4	4.4 ± 1.0	7.2 ± 0.5	8.5 ± 2.9	0.35		
Tau	10.2 ± 0.7	10.1 ± 0.3	18.2 ± 1.5	10.6 ± 0.3	0.03*		

Early treated groups (panel A) and late treated groups (panel B) at baseline (left) and at sacrifice (right). p values are from paired analysis between AR and AR+SIL

**Fig. 4:**
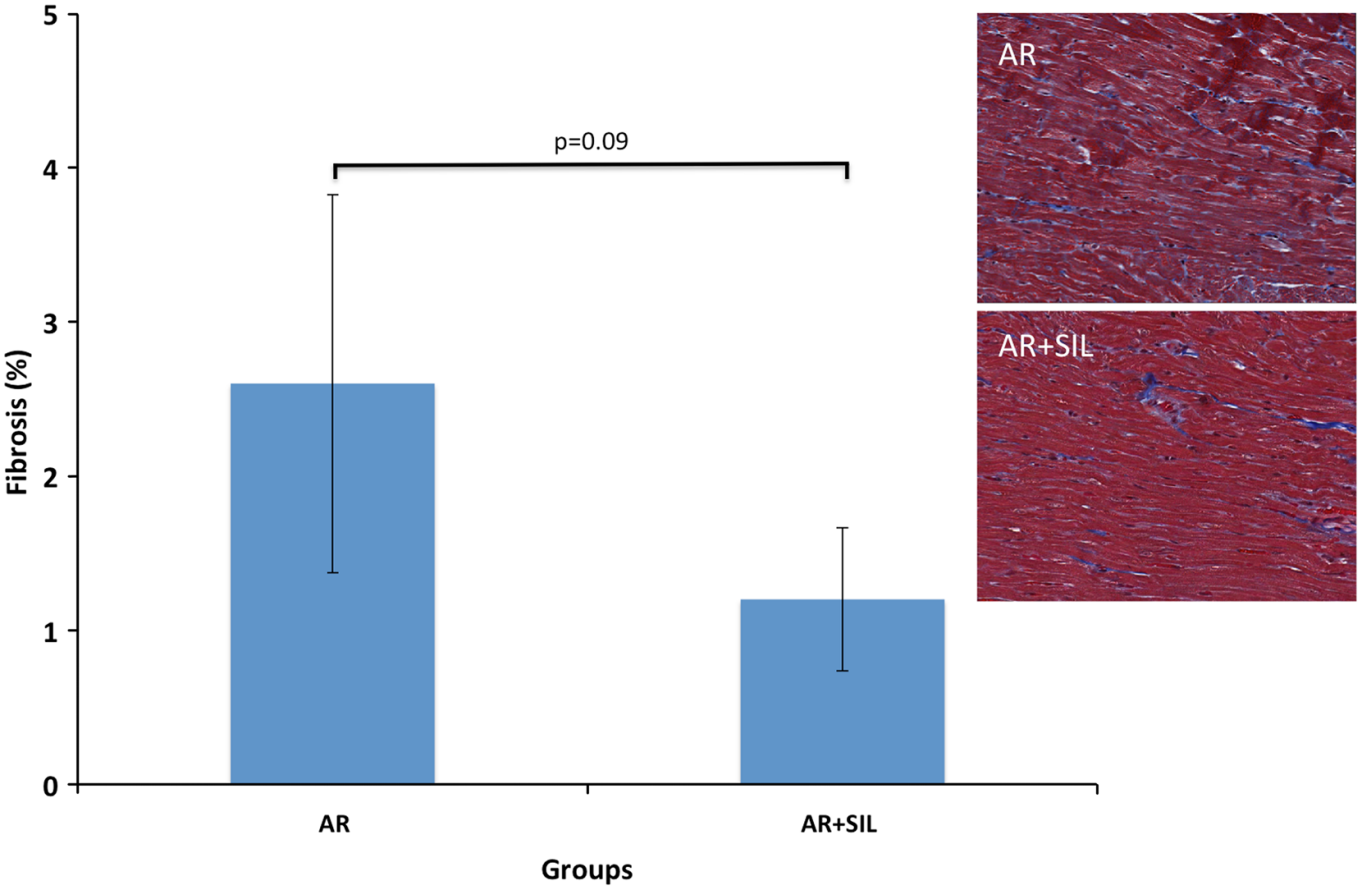
Fibrosis. *Bars* indicate degree of collagen fibrosis (%) in LV in untreated (AR) and treated (AR + SIL) rats. *Right* images show histological sections of LV tissue, with collagen *colored blue*

In the early treated group, histological analysis showed a borderline significant decrease in subendocardial collagen fibrosis compared to AR (early) (p = 0.09), Fig. 4.

**Fig. 5:**
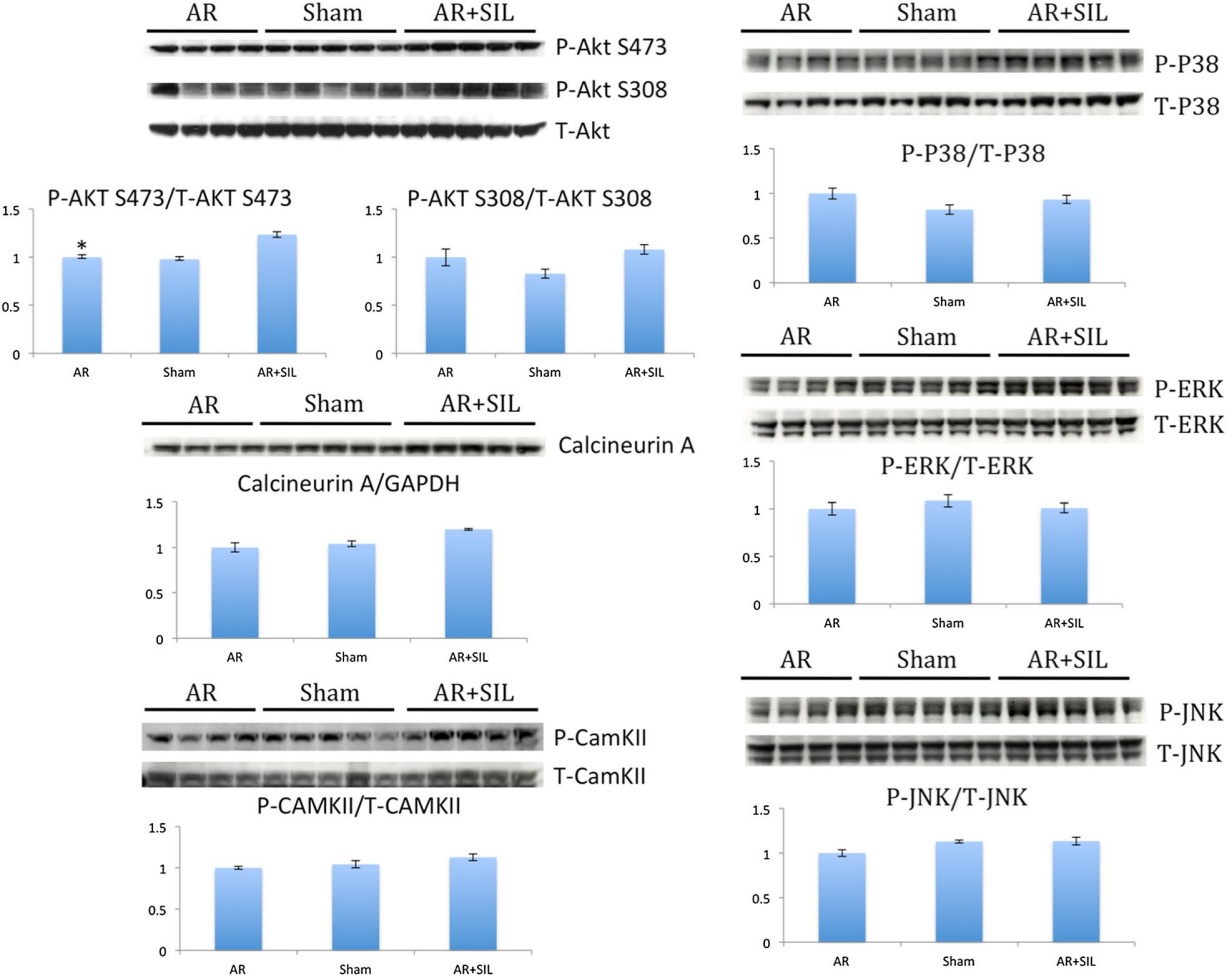
Protein analysis. *Bars* indicate the ratio of phosphorylated (P) to total (T) protein or housekeeping protein (GAPDH) normalized to untreated (AR). *p < 0.05 untreated (AR) vs. treated (AR + SIL)

We did not find any difference in phosphorylated levels of Akt S473 and Akt S308 between controls and sham–operated rats, while we found an increase in phosphorylated Akt S473 in AR + SIL compared to AR (p = 0.002). Total calcineurin and phosphorylated ratio of CamKII, ERK, JNK, and p38 were unchanged between groups, Fig. 5.

### Late treatment

Echocardiographic measurements of LV remodeling showed weaker effect of sildenafil treatment in the late treated group compared to the early treated. We did not find any significant differences in overall analysis of LV remodeling. However we saw a possible trend towards less wall thickness and LV mass (not shown) in AR + SIL (late) compared to AR (late) (p = 0.057 and p = 0.07, respectively). Echocardiography showed ventricular function to be unchanged by treatment (Fig. 3), as FS, SR and strain decrease was parallel in AR + SIL (late) and AR (late). This was confirmed by invasive hemodynamic measurements showing no difference in dp/dt_max_ and dp/dt_min_ in AR + SIL (late) compared to AR (late), Table 3. Diastolic blood pressure was positively affected by sildenafil treatment and thus PP was significantly lower in treated compared to untreated rats (46 ± 4 vs. 66 ± 7 mmHg, p = 0.05). As an isolated finding of improved LV diastolic function, we found Tau to be lower in treated compared to untreated rats.

## Discussion

In this study we demonstrate the effects of PDE5-inhibition in an experimental model of AR. We found PDE5-inhibition to blunt LV remodeling and LV dysfunction, when treatment was administered early. Although only borderline significant, the LV changes occurred along with a small reduction in subendocardial collagen fibrosis. Protein analysis showed increased levels of Akt in early treated rats while five known key pro–hypertrophic proteins were unaltered by treatment. In rats treated late with sildenafil, similar salutary effects were not noted. Our results indicate mild positive effects of PDE5-inhibition in experimental AR, however with a specific treatment-window wherein treatment is effective.

In humans the clinical course of AR is slow and more benign than other cardiac overload conditions (Toischer et al. 2010) and patients can be asymptomatic for years. Nonetheless, in some patients the balance between preload reserve, hypertrophy and increased afterload is ultimately exhausted and left untreated patients develop symptoms of congestive heart failure and eventually die (Maganti et al. 2010). Currently, the only definitive treatment is AVR (Bonow et al. 2008).

### Remodeling and LV function

Chronic AR causes an increase in end-diastolic volume and chamber compliance, while enlarged chamber size increases systolic wall stress. As disease progresses recruitment of preload reserve and compensatory hypertrophy enables LV to maintain a near-normal systolic function. This model of experimental AR compares to the long compensated phase in man, where there is a balanced eccentric LV and only subtle or no LV performance reduction.

In a model of transverse aortic constriction (TAC), early initiated sildenafil treatment has shown to inhibit development of LV hypertrophy and collagen fibrosis (Takimoto et al. 2005). Although our results were less pronounced, they are similar with the TAC model. We found sildenafil started before LV changes occurred to inhibit LV dilation. However, we did not observe LV diameters to return to normal or FS to improve dramatically. Additionally we observed sildenafil to only mildly reduce LV hypertrophy. Unlike the TAC model we did not find sildenafil to reverse already established LV changes.

Recently Kim et al. (2012) showed that anti-inflammatory and anti–apoptotic properties of sildenafil are playing an important role in preventing LV remodeling in a model of mitral valve regurgitation (MR). They found MR to activate stress responses like inflammatory pathways, DNA damage responses and cell cycle checkpoint pathways, all leading to LV remodeling. These negative effects were opposed by chronic treatment with sildenafil. Sildenafil attenuated remodeling by reversing dysregulated genes related to inflammation and apoptosis. Additionally they found sildenafil to down-regulate gene-sets related with hypertrophy. We did not specifically test anti-inflammatory and anti-apoptic properties, but we found an equivalent effect of sildenafil on remodeling.

### Signaling pathways

Due to lack of good models only little is known about intracellular hypertrophy-signaling in volume overload. Since AR consists of both volume and pressure overload components, we found the rationale for targeting hypertrophy-signaling pathways known to be operative in pure pressure and volume overload.

Cyclic GMP is a key second-messenger mediating an immense number of processes within the cardiovascular system, and dysfunction at any level of cGMP-signaling is closely related with cardiovascular disease (Tsai and Kass 2009). Takimoto et al. (Takimoto et al. 2005) have found that cardiac PDE5A expression is low under stable conditions and that PDE5A–inhibition shows little effect on the normal heart. On the contrary, PDE5A activity is increased during cardiac stress (Pokreisz et al. 2009) and this state is counter-balanced by increased PKG-1 activity. Takimoto et al. have shown cGMP catabolism to trigger several pro-hypertrophic signaling pathways resulting in pathological remodeling (Takimoto et al. 2005).

The PI3K–Akt pathway is a cascade affecting heart-growth and survival (Matsui and Nagoshi 2003). Physiologic stress activates Akt while sustained activation changes expression-profiling similar to pathological hypertrophy (Bernardo et al. 2010). Cyclic GMP has pleomorphic effects on Akt-activation. In rat ventricular myocytes Kato et al. (2005) found high-level cGMP signaling to induce apoptic cell death while lower levels were associated with cell survival. In our study we found Akt to be significantly increased in early treated rats. The level of increase observed is equal to protective effects of Akt when comparing to the many-fold increase seen in pathologic hypertrophy.

We found no significant difference in calcineurin between groups and thus no effect of sildenafil-treatment, which is in agreement with previously reports from an aortocaval-shunt model (Braun et al. 2004). Additionally, we did not observe any effect of sildenafil on CAMKII, p38, ERKs, and JNKs. This indicates that hypertrophy in AR is independent of these pathways.

Although our results suggest mild positive effects of sildenafil in AR the exact mechanism behind this is unknown. In our study several properties of sildenafil were left untested. One particular effect is worth mentioning. There is abundant PDE5 expression in lung tissue and PDE5–inhibition reduces pulmonary pressure and improves loading conditions (Lewis et al. 2007). Especially the ability of sildenafil to alleviate LV diastolic function has been investigated in clinical studies (Guazzi et al. 2007), but has recently shown disappointing results (Redfield 2013). However, sildenafil also reduces peripheral vascular resistance and blood pressure, which could reduce regurgitation-volume in AR by improving the pressure gradient between the aorta and the left ventricle during diastole. Additionally, reduction in afterload and wall stress alleviates left ventricular load and improves forward stroke volume. In combination these changes may translate into reduction in LV mass and preservation of LV function. However, our study was not designed to specifically test the vaso–active properties of sildenafil.

## Methods

### Animal model

AR was induced by echocardiography-guided closed-chest operation, described in detail elsewhere (Arsenault et al. 2002; Plante et al. 2003, 2004). Briefly, two aortic valve leaflets were punctured in a retrograde manner by a right–sided carotid arteriotomy. All rats were sedated with Isoflurane (1.5–2.0 % mixed with oxygen) followed by intra peritoneal cocktail (ketamine 90 mg kg^−1^ and Xylazine 10 mg kg^−1^) injection. Treatment with a PDE5A-inhibitor was initiated after 2 weeks of AR. Administration was performed by mixing sildenafil (Pfizer inc, Groton, CT, USA, 100 mg/kg/day) in transgenic dough–diet (Bioserv: Frenchtown, NJ, USA) that was fed to rats daily for a total of 10 weeks. All animal experiments were approved by the Johns Hopkins University Institutional Animal Care and Use Committee.

### Early treatment protocol

Twenty-four male Sprague–Dawley rats (age 19–20 weeks: Charles River: Wilmington, MA, USA) were divided into three groups: Rats with AR treated with sildenafil beginning at 2 weeks after AR (AR + SIL (early), n = 8), rats with severe AR receiving no treatment (AR (early), n = 8), and sham-operated rats receiving no treatment (Sham, n = 8). Sham-operated rats had all procedures performed except perforation of aortic valves. Rats were sacrificed 12 weeks after baseline examination.

### Late treatment protocol

Fifteen male Sprague–Dawley rats (age 9–10 weeks) were subjected to AR and divided into two groups: Rats with AR treated with sildenafil beginning at 12 weeks after AR [AR + SIL (late), n = 8], and rats with severe AR receiving no treatment [AR (late), n = 7]. Rats were sacrificed 22 weeks after baseline examination.

### Echocardiography

Echocardiography was performed on a Vivid 7 machine (GE Healthcare: Horton, Norway) with a 14 MHZ linear vascular probe. Examinations were performed at following time points: *Early study*: 0 (baseline), 2, 6, 9 and 12 weeks after AR. *Late study*: 0, 2, 6, 9, 10, 12, 16, 19, and 22 weeks after AR.

Diameters, wall thickness (WT), and shortening fraction (FS) were assessed by M–mode. Measurements were averaged from four beats, two parasternal long–axis (PLAX) and two short–axis (SAX) planes. FS was calculated as [LVEDD − LVESD]/[LVEDD]. As a measure of WT, septum and LV posterior walls were averaged. Speckle tracking was performed by acquiring 2D cine loops from at least six cardiac cycles, in mid-ventricular SAX plane at frame rates >70 s^−1^. Tracking was performed by using at least three consecutive cardiac cycles in EchoPAC’s strain modality analysis software (v. 7.0, GE Healthcare, Waukesha, WI, USA). The global trace representing averaged circumferential deformation was used. For strain-rate measurements the global traces were used.

### Hemodynamic measurements

Invasive LV hemodynamic measurements were performed at baseline and before sacrifice. In anesthetized rats a micro–tip conductance pressure–volume catheter (Millar Instruments, Inc.: Houston, TX, USA) was inserted into the heart by right–sided carotid arteriotomy. Pressure–volume loops (PV-loops) were obtained under resting conditions.

### Histology

Formalin (10 %) fixed and paraffin embedded myocardium from LV was sectioned into 5 µm thick slices and stained with H&E and Masson’s trichrome. For analysis, four transmural areas from each circumferential LV-section (one from each rat) were extracted using Scanscope software (Aperio Technologies inc.: Vista, CA, USA). For fibrosis quantification FRIDA (FRamework for Image Dataset Analysis: The Johns Hopkins University, Baltimore, MD, USA) was used. This is a custom open-source image analysis software package for color image datasets. Epicardium, pericardium and larger vessels were excluded from this analysis.

### Protein analysis

Tissue samples from snap–frozen hearts were prepared for protein analysis as described elsewhere (Champion et al. 2004). Proteins were prepared by standard procedures for Western blotting and antibody probation. NIH Image J software was used for quantifying blots (NIH, version 1.45b). Phospho-protein activation was quantified as ratio of phosphorylated protein to total protein and GAPDH.

### Statistical analysis

AR was documented by comparing sham-operated and untreated (AR) rats. The effect of treatment was documented by comparing medically treated (AR + SIL) with untreated (AR) rats. The difference between multiple groups was analyzed with ANOVA and Tukey’s test for multiple comparisons. Serial echocardiography was compared by repeated measures. All p values are two-tailed and a significance level of 0.05 was used. Statistics are given as mean ± standard error unless stated otherwise. All analysis were performed using SAS^®^ software (SAS for windows, release 9.1, SAS Institute Inc., Cary, NC, USA).

## Limitations

Experimental AR is different to what is seen clinically, mainly due to the acute nature of the model. However this is a limitation in most experimental valve models and was accounted for by postponing measurements until after the acute phase, which allowed adaptation to the new loading conditions (Olsen et al. 2013).

Ten weeks of medical treatment is similar to what has been previously used (Takimoto et al. 2005) and addresses the early chronic phase of AR. However longer treatment-duration could reveal more chronic effects of sildenafil.

Our sample size is modest but adequate to demonstrate significant differences in the early treatment group. A larger sample size may have revealed additional significant effects.

As in clinical settings, speckle tracking imaging faces limitations with frame rates, which is amplified with fast heart rates. However, in small animals the shortened diastolic filling time is followed by a systolic phase that is as long or even longer, which allows >7 frames to reconstruct the systolic part of the strain curve. This number is similar to what is used in human MR tagging studies.

## Conclusion

In a rat model of chronic severe AR, we found sildenafil to mildly inhibit left ventricle remodeling and systolic dysfunction when treatment was started early in the course of disease.
